# Detection of polyreactive immunoglobulin G facilitates diagnosis in children with autoimmune hepatitis

**DOI:** 10.1007/s12072-024-10695-1

**Published:** 2024-07-08

**Authors:** Bastian Engel, Jana Diestelhorst, Katharina Luise Hupa-Breier, Theresa Kirchner, Nicole Henjes, Stephanie Loges, Muhammed Yuksel, Wojciech Janczyk, Claudine Lalanne, Kalliopi Zachou, Ye H. Oo, Jérôme Gournay, Simon Pape, Joost P. H. Drenth, Amédée Renand, George N. Dalekos, Luigi Muratori, Piotr Socha, Yun Ma, Cigdem Arikan, Ulrich Baumann, Michael P. Manns, Heiner Wedemeyer, Norman Junge, Elmar Jaeckel, Richard Taubert

**Affiliations:** 1 Department of Gastroenterology, Hepatology, Infectious Diseases and Endocrinology, Hannover Medical School, Carl-Neuberg-Straße 1, 30625 HepatologyHannover, Germany; 2 Division of Pediatric Gastroenterology and Hepatology, Department of Pediatric Nephrology, Hepatology and Metabolic Disorders, Hannover Medical School, Hannover, Germany; 3 Institute of Liver Studies, Department of Inflammation Biology, School of Immunology and Microbial Sciences, King’s College Hospital, King’s College London, London, UK; 4 Koç University Research Centre for Translational Medicine (KUTTAM)-Liver Immunology Lab, Istanbul, Turkey; 5 Department of Biomedical Sciences, College of Liberal Arts and Life Sciences, University of Westminster, London, UK; 6 Department of Gastroenterology, Hepatology, Nutritional Disorders and Pediatrics, The Children’s Memorial Health Institute, Warsaw, Poland; 7 Department of Medical and Surgical Sciences, University of Bologna, Bologna, Italy; 8 Institute of Internal Medicine and Hepatology, Larissa, Greece; 9 l University Hospital of Larissa, Larissa, Greece; 10 Centre for Liver and Gastro Research, National Institute of Health Research Birmingham Biomedical Research Centre, Institute of Immunology and Immunotherapy, The Medical School, Birmingham, United Kingdom & Liver transplant and Hepatobiliary Unit, University Hospital Birmingham NHS Foundation Trust, Birmingham, UK; 11 Institut Des Maladies de L’Appareil Digestif (IMAD), Nantes Université, CHU Nantes, Hépato-Gastro-Entérologie, Inserm CIC 1413, 44000 Nantes, France; 12 Department of Gastroenterology and Hepatology, Radboud University Medical Center, Nijmegen, The Netherlands; 13 Center for Research in Transplantation and Translational Immunology, Nantes Université, UMR 1064, Inserm, 44000 Nantes, France; 14 Department of Pediatric Gastroenterology, Hepatology, and Nutrition, Koç University School of Medicine, Istanbul, 34010 Turkey; 15 European Reference Network on Hepatological Diseases (ERN RARE-LIVER), Hamburg, Germany; 16 Oncology, Hemostaseology and Palliative Care, Johannes Wesling Medical Center Minden, University Clinic for Haematology, UKRUB, University of Bochum, Minden, Germany; 17 Ajmera Transplant Center, Toronto General Hospital, United Health Network, University of Toronto, Toronto, Canada

**Keywords:** Antinuclear antibody, Anti-smooth muscle antibody, Anti-liver kidney microsomal antibody, Anti-soluble liver antigen/liver pancreas, Huntingtin-interacting protein 1-related protein (HIP1R)

## Abstract

**Objective:**

The detection of autoantibodies is essential to diagnose autoimmune hepatitis (AIH). Particularly in children, specificity of autoantibodies decreases due to lower titers being diagnostic and being present not only in AIH but also in other liver diseases. Recently, quantification of polyreactive IgG (pIgG) for detection of adult AIH showed the highest overall accuracy compared to antinuclear antibodies (ANA), anti-smooth muscle antibodies (anti-SMA), anti-liver kidney microsomal antibodies (anti-LKM) and anti-soluble liver antigen/liver pancreas antibodies (anti-SLA/LP). We aimed to evaluate the diagnostic value of pIgG for pediatric AIH.

**Design:**

pIgG, quantified using HIP1R/BSA coated ELISA, and immunofluorescence on rodent tissue sections were performed centrally. The diagnostic fidelity to diagnose AIH was compared to conventional autoantibodies of AIH in training and validation cohorts from a retrospective, European multi-center cohort from nine centers from eight European countries composed of existing biorepositories from expert centers (*n* = 285).

**Results:**

IgG from pediatric AIH patients exhibited increased polyreactivity to multiple protein and non-protein substrates compared to non-AIH liver diseases and healthy children. pIgG had an AUC of 0.900 to distinguish AIH from non-AIH liver diseases. pIgG had a 31–73% higher specificity than ANA and anti-SMA and comparable sensitivity that was 6–20 times higher than of anti-SLA/LP, anti-LC1 and anti-LKM. pIgG had a 21–34% higher accuracy than conventional autoantibodies, was positive in 43–75% of children with AIH and normal IgG and independent from treatment response.

**Conclusion:**

Detecting pIgG improves the diagnostic evaluation of pediatric AIH compared to conventional autoantibodies, primarily owing to higher accuracy and specificity.

**Graphical Abstract:**

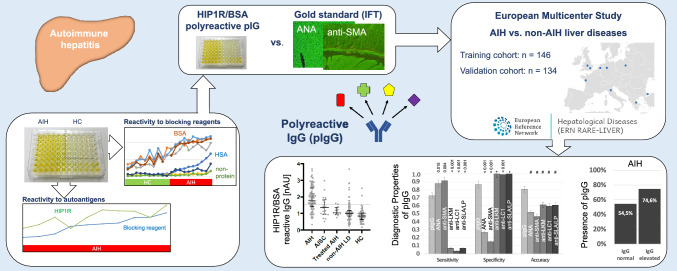

**Supplementary Information:**

The online version contains supplementary material available at 10.1007/s12072-024-10695-1.

## Introduction

Autoimmune hepatitis (AIH) is a rare chronic inflammatory liver disease that affects people of all age groups with female predominance and a rising incidence [1]. The prevalence was reported at 19.44 per 100 000 inhabitants in Europe [2]. AIH mostly needs to be treated by immunosuppressive therapy and a lack of treatment response as defined by persistently elevated transaminases and immunoglobulin G as well as the occurrence of cirrhosis amongst other factors are related to an unfavourable outcome [3–5].

The diagnosis is based on elevated liver enzymes, positivity for autoantibodies, elevation of immunoglobulin G (IgG), compatible or typical findings in liver histology and exclusion of other causes of liver disease. The presence of extrahepatic autoimmunity and/or a family history of autoimmune diseases increases the probability of having AIH. Typical histological findings consist mainly of a combination of interface hepatitis, plasma cell-rich infiltrates, emperipolesis and rosette formation [6, 7]. However, all four features were found in only 56% of children with AIH and histological findings are not always conclusive, especially in early stages of the disease [8]. Recently, new criteria were proposed for the histopathological diagnosis of AIH, in addition to the aforementioned serological features, but these are pending validation [9]. Furthermore, there are many diseases which manifest in childhood and can be misdiagnosed as AIH [10–12]. Testing for autoantibodies on rodent tissue sections of liver, kidney and stomach using immunofluorescence remains a major component for the diagnosis of AIH and is endorsed by the international autoimmune hepatitis group (IAIHG) [13, 14]. This approach is time consuming and requires expertise. With the detection of target antigens, antigen-specific detection of autoantibodies using ELISA is frequently applied. Antinuclear antibodies (ANA), anti-smooth muscle antibodies (anti-SMA) and/or anti-soluble liver antigen/liver pancreas antibodies (anti-SLA/LP) characterize the more prevalent AIH type 1 while anti-liver kidney microsomal antibodies (anti-LKM) or anti-liver cytosol antibodies type 1 (anti-LC1) define type 2 with a very recent study suggesting that liver-related outcome is not different between both types [13, 15]. A meta-analysis evaluated the diagnostic capabilities of ANA, anti-SMA and anti-SLA/LP including both children and adults [16]. ANA had moderate sensitivity and specificity (65% and 75%), anti-SMA had moderate sensitivity and good specificity (59% and 93%) and anti-SLA/LP had low sensitivity and very good specificity (19% and 99%) [16]. However, the authors noted a lack of consistency arguing for necessity of expertise in evaluating autoantibodies using immunofluorescence which is supported by recent studies on patients with autoimmune liver diseases that compared test performance between various test systems [17, 18]. We recently published that IgG in adult AIH patients has polyreactivity to a variety of human and non-human antigens [19]. We showed that quantification of this polyreactive IgG (pIgG) can be used as a diagnostic test to distinguish AIH from non-AIH liver disease (non-AIH LD) with a better specificity than ANA and anti-SMA and the highest overall accuracy compared to all conventional autoantibodies [19, 20].

The aim of the current study was to assess the diagnostic performance of pIgG in comparison to conventional autoantibodies in a multi-center cohort of children with AIH, non-AIH LD and healthy controls (HC).

## Patients and methods

### Patients

After the initial establishment of the pIgG assay in adult patients at our center [19], a pediatric cohort was retrospectively recruited from our local biomaterial repository at Hannover Medical School (Hannover, Germany) as a training cohort for testing the assay performance of the pIgG ELISA in children. A pediatric validation cohort was retrospectively recruited from existing biomaterial repositories and data bases from eight centers from seven European countries following a call for participation to European Reference Network (ERN) rare-liver and IAIHG members after EASL international liver congress 2018 in Paris. Children were included if they were younger than or just recently turned 18 years at the time of sampling and were cared for by the local pediatricians. For both cohorts, all children with available serum samples and signed informed consent by the children’s parents or themselves as applicable were included.

### Diagnostic criteria

For both cohorts, criteria for AIH were a score compliant with a least “probable AIH” after performance of a liver biopsy according to the currently proposed scoring system and clinical follow-up including treatment response [6, 21]. All samples from children with untreated AIH were taken at diagnosis before initiation of immunosuppressive therapy, because pIgG declined under therapy in adult AIH patients [19].

Non-AIH liver diseases were diagnosed according to current guidelines [6, 22–25] and for alpha-1 antitrypsin deficiency by reduced serum alpha-1 antitrypsin and genotyping. All non-AIH liver diseases control samples were taken from patients not on immunosuppressive therapy at the time of sampling. The group of non-AIH liver diseases was mostly comprised of children with primary sclerosing cholangitis, metabolic dysfunction-associated steatotic liver disease/metabolic dysfunction-associated steatohepatitis (MASLD/MASH), drug-induced liver injury (DILI), hereditary/metabolic diseases (alpha-1 antitrypsin deficiency, Wilson’s disease and progressive familial intrahepatic cholestasis) as well as single rare causes of hepatitis grouped as “other” (Suppl. Figure 2). For the validation cohort, additional samples from children on immunosuppressive therapy were available for analysis. In adults, blood donors are regarded as HC, which cannot be transferred to children. Thus, blood samples of patients with mostly functional gastrointestinal disorders based on Rome IV criteria [26] served as HC. These children tested negative for celiac disease-related autoantibodies.

### Conventional autoantibodies

Testing for conventional autoantibodies with indirect immunofluorescence (IIF) or ELISA was performed centrally at Hannover Medical School according to the current guidelines [13, 14, 27].

### Quantification of polyreactive immunoglobulin G

Quantification of pIgG was performed as recently published [19] using an ELISA measuring reactivity of patients IgG to huntingtin-interacting protein 1-related protein (HIP1R) and bovine serum albumin (BSA).

### Ethics

The study was approved by the ethical committee at Hannover Medical School (MHH Ethikkommission, Hannover, Germany; approval numbers 1025–2011 and 2665–2015). Written informed consent was obtained from parents and children at all centers as applicable. The use of retained samples from clinical laboratories at Hannover Medical School from pediatric patients with liver diseases was approved by the local ethical committee (approval number 2817–2015).

The respective local ethical committees approved the use of material and data from external patients.

The study conforms to the ethical guidelines of the 1975 Declaration of Helsinki as reflected in a priori approval by the institution’s human research committee. All experiments were performed in accordance with relevant guidelines and regulations.

Further methods are outlined in the supplemental data file.

## Results

### Exploration of polyreactivity of IgG from children with AIH

**Fig. 1:**
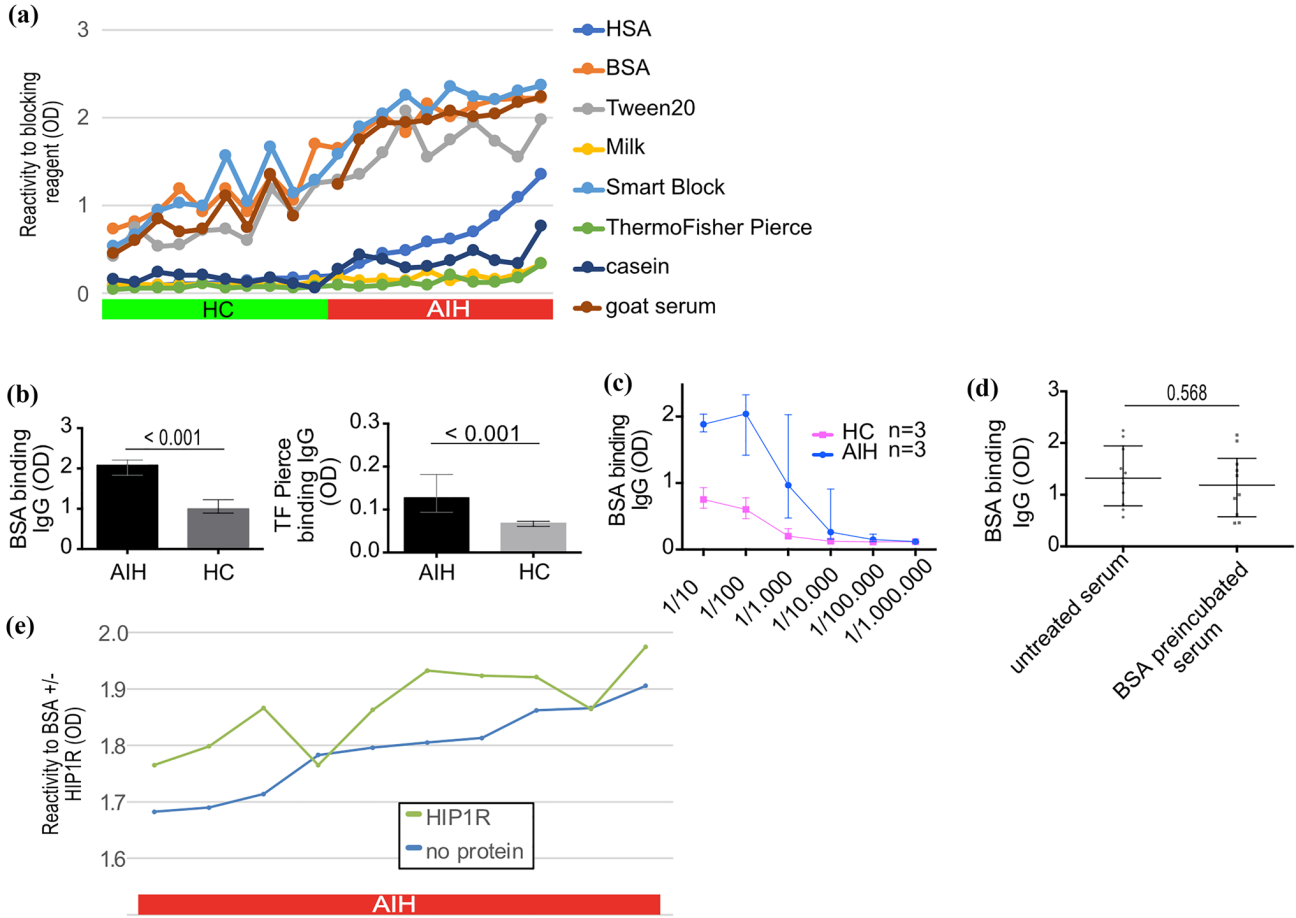
IgG from children with autoimmune hepatitis is polyreactive. **A** Reactivity of IgG from children with untreated AIH and HC to multiple blocking reagents. HSA: human serum albumin, *BSA* bovine serum albumin, OD: optical density. **B** IgG reactivity to blocking reagent (median and IQR; Mann–Whitney-U test). TF: ThermoFisher. **C** Decrease of reactivity to BSA in children with AIH and HC following serial dilution. **D** Maintained BSA reactive IgG after BSA liquid–liquid preincubation of sera from children with AIH (Mann–Whitney-*U* test). **E** Reactivity of IgG from children with AIH to BSA (blue line) or BSA/HIP1R (green line) (colour figure online)

Polyreactivity of IgG to multiple ELISA blocking reagents was detected in sera from children with untreated AIH (Fig. 1a) using an ELISA without coating of a target antigen. As pIgG declines under therapy in adults, we initially focussed our efforts on untreated pediatric patients with AIH [19]. The highest binding was found for BSA while the lowest binding was found for Pierce™ Protein-Free Blocking Buffer (TF Pierce) from ThermoFisher. Irrespective of the magnitude of binding, sera from children with AIH exhibited higher binding than HC (shown for BSA and TF Pierce in Fig. 1b). Serial dilution decreased the absorbance of BSA binding IgG in children with AIH and HC (Fig. 1c). The difference between AIH and HC vanished only at very high dilutions of ≥ 1/10.000. Liquid–liquid preincubation of sera from children with AIH (*n* = 10) with excess BSA did not affect the absorbance of BSA binding IgG in these patients (Fig. 1d). Addition of HIP1R to the ELISA as a target antigen increased the absorbance compared to BSA alone in children with untreated AIH (Fig. 1e).

As IgG from children with untreated AIH exhibited similar polyreactivity as published in adults [19], we continued the evaluation of pIgG in a multi-center cohort of children with AIH, non-AIH liver disease (non-AIH LD) and HC.

### Comparison of candidate autoantigens to quantify pIgG in children

Overall, 285 serum samples were available for analysis (84 untreated AIH, 13 AIH on immunosuppressive therapy, 19 autoimmune sclerosing cholangitis (AISC), 119 non-AIH LD, 50 HC). The cohort from our center in Hannover was used as a training cohort and samples from the other eight centers were used as external validation cohort (Table 1, Suppl. Table 1).

**Table 1:** Clinical data of children with AIH and non-AIH liver disease

	Training cohort			Validation cohort		
	Untreated AIH	AISC	Non-AIH liver disease	Untreated AIH	AISC	Non-AIH liver disease
Number	53	8	51	31	11	68
Age (median (range))	12 (3—17)	14 (7—16)	12 (0—17)	12 (2—18)	14 (5—17)	12 (1—18) (n = 66)
Female sex, n (%)	38 (71.7)	5 (62.5)	17 (33.3)	16 (51.6)	1 (9.1)	31 (45.6)
AST, median [times upper limit of normal] (range)	10.9 (1.1—114.1)	2.4 (1.0—5.8)	2 (0.7—50.9) (n = 50)	11.7 (0.5—42.3)	6.8 (0.6—29.0)	0.9 (0.4—152.4) (n = 62)
ALT, median [times upper limit of normal] (range)	12.3 (1.0—84.9)	3.1 (0.4—4.4)	2.5 (0.3—40.2) (n = 50)	9.0 (1.4—43.7) (n = 22)	2.8 (1.0—5.9) (n = 4)	1.4 (0.2—216.6) (n = 61)
IgG, median [times upper limit of normal] (range)	1.6 (0.5—6.1)	1.3 (0.8—2.0) (n = 6)	0.9 (0.5—1.8) (n = 45)	1.5 (0.6—3.3) (n = 29)	1.2 (0.5—2.6)	0.6 (0.3—1.2) (n = 43)
Seronegative AIH, n (%)	0 (0)	na	na	1 (3.2)	na	na
AIH-1, n (%)	49 (92.4)	na	na	27 (87.1)	na	na
AIH-2, n (%)	4 (7.6)	na	na	4 (12.9)	na	na

Longer storage duration increased reactivity of pIgG and it differed slightly, depending on the respective center [19] (Suppl. Figure 1) as described in adults [19]. Therefore, all samples were normalized for storage duration and center background of non-AIH LD and HC as published for adults and outlined in the Supplemental methods section (Suppl. Figure 1) [19].

**Fig. 2:**
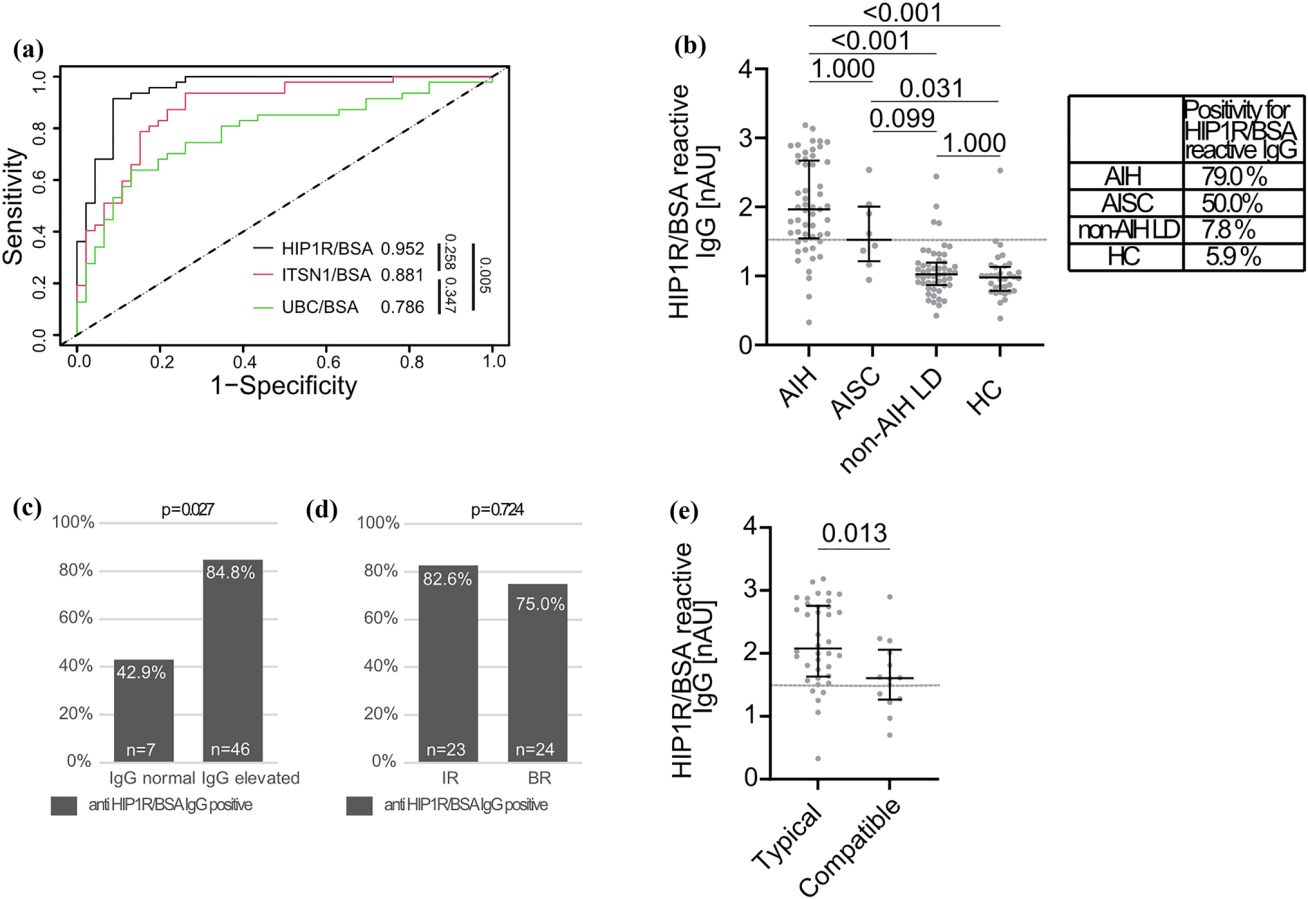
Reactivity of anti-HIP1R/BSA in the training cohort. **A** AUC of anti-HIP1R/BSA (black line), anti-UBC/BSA (green line) and anti-ITSN1/BSA (red line) to diagnose AIH (n = 46) (p values for respective pair-wise comparisons; DeLong test). **B** anti-HIP1R/BSA reactive IgG in AIH (*n* = 53), AISC (*n* = 8), non-AIH LD (*n* = 51) and HC (*n* = 34). (grey line: cut-off 1.5nAU, median and IQR, Kruskal–Wallis test with Bonferroni’s post-hoc test). Proportions of anti-HIP1R/BSA positive children are summarized in the box. Proportion of children with AIH positive for anti-HIP1R/BSA with **C** normal or elevated IgG and **D** incomplete response (IR) or biochemical remission (BR) (Fisher's Exact test). **E** Anti-HIP1R/BSA reactive IgG in children with AIH and typical (*n* = 38) or compatible histology (*n* = 14) (Mann–Whitney *U* test). HIP1R/BSA: huntingtin-interacting protein 1-related protein/bovine serum albumin; ITSN1/BSA: intersectin 1/bovine serum albumin; UBC/BSA: ubiquitin/bovine serum albumin (colour figure online)

During the discovery of polyreactive binding of IgG in untreated adult patients with AIH, multiple autoantigens were recognized by patient IgG on a commercial protein microarray including HIP1R, UBC and ITSN1 [19]. To assess the best autoantigen for the quantification of polyreactivity in children, all three autoantigens were tested head-to-head to assess the respective diagnostic fidelity using AUC in the training cohort. Forty-seven children with AIH and 46 children with non-AIH LD with measurements of all three markers were available (Suppl. Table 2). AUC was highest for HIP1R/BSA (AUC: 0.952) which was superior to UBC/BSA (AUC: 0.786, *p* = 0.005) and comparable to ITSN1/BSA (AUC: 0.881, *p* = 0.258) (Fig. 2a). Even though the difference between ITSN1/BSA and HIP1R/BSA was not statistically significant we continued with HIP1R/BSA because of the highest AUC and for concise comparison with results from adult patients [19].

### HIP1R/BSA reactive IgG for the diagnosis of AIH: training cohort

Clinical data of children within the training cohort are summarized in Table 1 and Suppl. Table 1. Children with AIH exhibited higher levels of HIP1R/BSA binding IgG compared to children with non-AIH LD and HC, while no differences were observed between the latter. Patients with AISC were regarded separately as AISC typically exhibits features of both AIH and sclerosing cholangitis and necessitates immunosuppressive treatment. A cut-off of 1.5 nAU was identified for optimal discrimination between AIH and non-AIH LD guided by the Youden’s index (AUC 0.900, *p* < 0.001, *n* = 104). This cut-off was marginally lower if AISC patients were included into the AIH group with a comparable AUC (0.890, *p* < 0.001) (Suppl. Table 3). As the cohort was recruited to discriminate AIH from non-AIH LD the cut-off of 1.5 nAU was used for further analyses. In total, 79% of children with AIH had HIP1R/BSA reactive IgG above the cut-off of 1.5 nAU while only 7.8% of non-AIH LD and 5.9% of HC had positive results for HIP1R/BSA reactive IgG. Median levels of HIP1R/BSA reactive IgG were significantly higher in patients with AIH than non-AIH LD and HC (p < 0.001 respectively) (Fig. 2b). Patients with AISC trended towards higher levels of HIP1R/BSA reactive IgG compared to non-AIH LD (*p* = 0.099) (Fig. 2b; Suppl. Figure 2a). Anti-HIP1R/BSA was equally sensitive to ANA and anti-SMA to diagnose AIH while specificity was superior. Sensitivity was higher for anti-HIP1R/BSA compared to anti-LKM and anti-SLA. Anti-HIP1R/BSA had the highest overall accuracy compared to the conventional autoantibodies (Table 2). Even when applying the higher cut-off of 1:80 that attributes two points (in contrast to one point for ANA and anti-SMA equal or above 1:20) in the used scoring system, anti-HIP1R/BSA maintains superior specificity while being equally sensitive as ANA and anti-SMA (Suppl. Table 4). In contrast to the previously published adult cohort [19], the present pediatric cohort did not contain autoantibody negative AIH patients with the diagnostic cut-off of 1:20. To assess the dependency of pIgG on conventional autoantibodies, children with pAIH were grouped according to the presence of ANA and anti-SMA. Median pIgG levels were not different in children with positivity for ANA in comparison to children without ANA (2.00 vs. 1.73 nAU; *p* = 0.127) as well as in children with positivity for anti-SMA in comparison to children without anti-SMA (1.97 vs. 1.84 nAU; *p* = 0.669). Five children had AIH type 2 and were positive for anti-LKM (*n* = 3) or anti-LC1 (*n* = 2) of which three were positive for anti-HIP1R/BSA (60%). Three children with AIH were positive for anti-SLA/LP of which all were positive for anti-HIP1R/BSA (100%). Five children had seronegative AIH with a cut-off of 1:80 for ANA and anti-SMA. Of these five children four were positive for anti-HIP1R/BSA (80%).

**Table 2:**
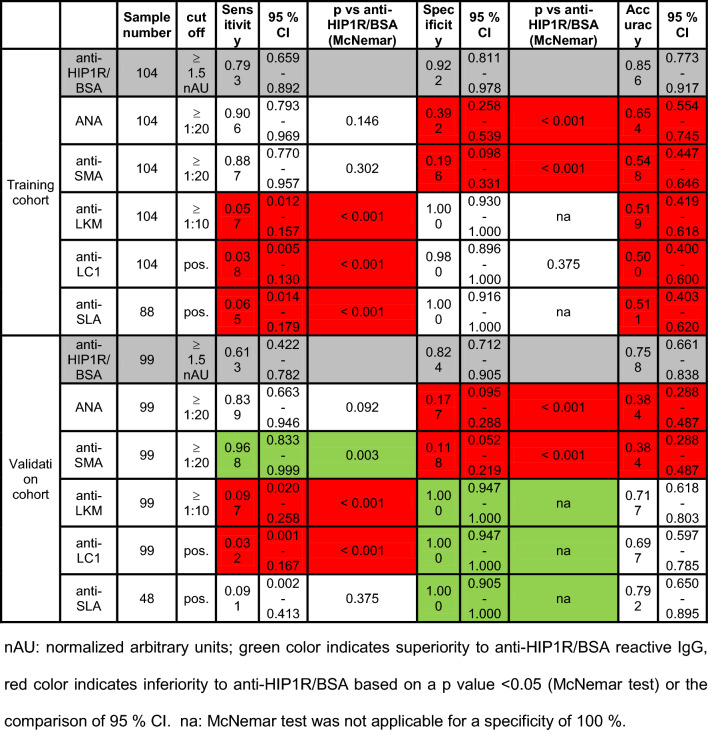
Diagnostic test performance of different antibodies to distinguish AIH from non-AIH liver disease

Polyreactive IgG correlated with the total amount of IgG in children with AIH (Spearman’s rank-correlation coefficient (SR) = 0.489, *p* < 0.001, *n* = 53) and the sensitivity of anti-HIP1R/BSA was lower in children with normal IgG (42.9%, *n* = 7) compared to those with elevated total IgG (84.8%, *n* = 46) (*p* = 0.027) (Fig. 2c). Anti-HIP1R/BSA did not correlate with ALT (SR = 0.124, *p* = 0.377, *n* = 53) or age (SR = 0.004, *p* = 0.975, *n* = 53) in children with AIH. Median levels were not different between boys and girls with AIH (1.90 vs. 1.98 nAU, *p* = 0.643). Positivity for anti-HIP1R/BSA at diagnosis was not predictive of subsequent therapy response of the immunosuppressive therapy (Fig. 2d). Median pIgG levels were higher in children with a typical histopathology of AIH than in children with a compatible histopathology (2.08 vs. 1.60 nAU; *p* = 0.013; Fig. 2e). Median pIgG levels did not differ if children were grouped based on the presence or absence of plasma cells, interface hepatitis or emperipolesis (Suppl. Figure 3a).

### HIP1R/BSA reactive IgG for the diagnosis of AIH: external validation cohort

**Fig. 3:**
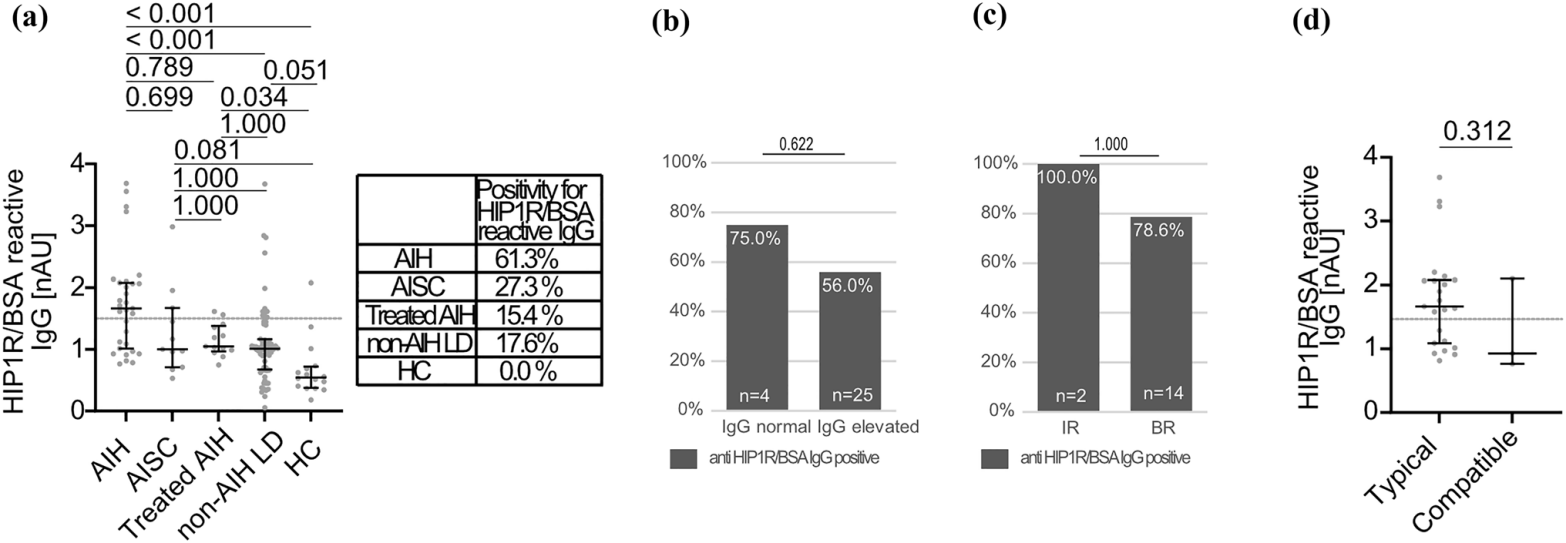
Reactivity of anti-HIP1R/BSA in the validation cohort. **A** anti-HIP1R/BSA reactive IgG in AIH (*n* = 31), AISC (*n* = 11), AIH on treatment (*n* = 13), non-AIH LD (*n* = 68) and HC (*n* = 16) (grey line: cut-off 1.5nAU, median and IQR, Kruskal–Wallis test with Bonferroni’s post-hoc test). Proportions of anti-HIP1R/BSA positive children within the respective disease groups are summarized in the box. Proportion of children with AIH with positivity for anti-HIP1R/BSA with **B** normal or elevated IgG and **C** incomplete response (IR) or biochemical remission (BR) (Fisher’s Exact test). **D** Anti-HIP1R/BSA reactive IgG in children with AIH and typical (*n* = 23) or compatible histology (*n* = 3) (Mann–Whitney *U* test). *HIP1R* huntingtin-interacting protein 1-related protein, *BSA* bovine serum albumin (colour figure online)

Clinical patient characteristics of the validation cohort are summarized in Table 1 and Suppl. Table 1. Children with AIH had higher levels of HIP1R/BSA binding IgG than children with non-AIH LD and HC. Positivity for anti-HIP1R/BSA was present in 61.3% of children with AIH, 17.6% of children with non-AIH LD (*p* < 0.001 vs. AIH) and none of the HC (*p* < 0.001 vs. AIH) (Fig. 3a). Children with AISC had comparable levels of anti-HIP1R/BSA compared to other non-AIH LD but statistical significance to both AIH and non-AIH LD was missed (Fig. 3a). Children with drug-induced liver injury (DILI, n = 3; Suppl. Table 5) trended towards higher levels than other non-AIH LD within the range of children with AIH (Suppl. Figure 2b). Anti-HIP1R/BSA was equally sensitive to ANA and less sensitive than anti-SMA while being more specific than both. Sensitivity was better for anti-HIP1R/BSA than for anti-LKM and anti-LC1. Specificity of anti-LKM, anti-LC1 and anti-SLA/LP was superior to anti-HIP1R/BSA. Accuracy of pIgG was superior to ANA and anti-SMA and comparable to anti-LKM, anti-LC1 and anti-SLA/LP (Table 2). This remained when applying the cut-off of 1:80 for ANA and anti-SMA (Suppl. Table 4). Median pIgG levels were not different in children with positivity for ANA in comparison to children without ANA (1.67 vs. 1.67 nAU; *p* = 0.480) as well as in children with positivity for anti-SMA in comparison to children without anti-SMA (1.67 vs. 1.67 nAU; no statistical test applicable with only one child in the anti-SMA-negative group). Four children had AIH type 2 with anti-LKM antibodies or anti-LC1 antibodies and one had anti-SLA/LP antibodies. All were negative for anti-HIP1R/BSA.

Polyreactive IgG did not correlate with total IgG (SR = 0.196, *p* = 0.308, *n* = 29), ALT (SR = − 0.188, *p* = 0.403, *n* = 22) or age (SR = 0.238, *p* = 0.197, *n* = 31). Median levels of anti-HIP1R/BSA were not different between boys and girls with AIH (1.64 nAU vs. 1.73 nAU; *p* = 0.740). Sensitivity of pIgG did not differ statistically whether IgG was elevated or not (56% vs 75%, p = 0.622) (Fig. 3b). Positivity for anti-HIP1R/BSA did not predict subsequent treatment response of immunosuppressive therapy (Fig. 3c). One child had seronegative AIH and was positive for anti-HIP1R/BSA with a cut-off of 1:20 for ANA and anti-SMA. Two children had seronegative AIH if a cut-off of 1:80 was used of which one was positive for anti-HIP1R/BSA. Median pIgG levels were not different in children with a typical histopathology of AIH than in children with a compatible histopathology (1.67 vs. 0.93 nAU; p = 0.312; Fig. 2e). Median pIgG levels did not differ if children were grouped based on the presence or absence of plasma cells, interface hepatitis or emperipolesis (Suppl. Figure 3b).

### HIP1R/BSA reactive IgG for the diagnosis of AIH: overall diagnostic fidelity

**Fig. 4:**
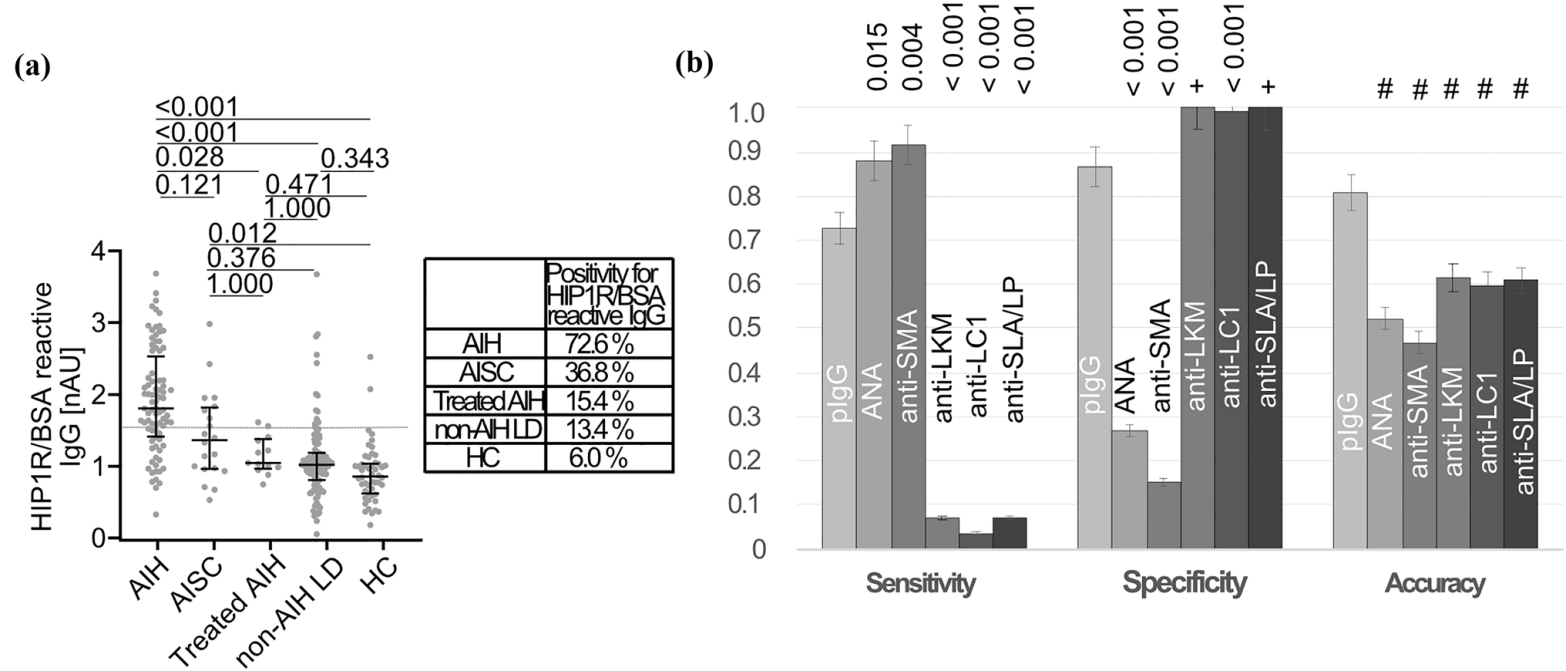
Reactivity of anti-HIP1R/BSA in the overall cohort. **A** anti-HIP1R/BSA reactive IgG in AIH (*n* = 84), AISC (*n* = 19), AIH on treatment (*n* = 13), non-AIH LD (*n* = 119) and HC (*n* = 50) (grey line: cut-off 1.5nAU, median and IQR, Kruskal–Wallis test with Bonferroni’s post-hoc test). Proportions of anti-HIP1R/BSA positive children within the respective disease groups are summarized in the box. **B** Diagnostic properties of polyreactive IgG (pIgG) in comparison to conventional autoantibodies (cut-off 1:20 for ANA and anti-SMA, cut-off 1:10 for anti-LKM) of AIH (McNemar test in case p-value is shown; + McNemar test not applicable, superiority to pIgG by comparison of 95% CI; # inferiority to pIgG by comparison of 95% CI) *HIP1R* huntingtin-interacting protein 1-related protein, *BSA* bovine serum albumin, *ANA* antinuclear antibodies, *anti-SMA* anti-smooth muscle antibodies, anti-SLA/LP anti-soluble liver antigen/liver-pancreas antibodies, *anti-LKM* anti-liver kidney microsomal antibodies, *anti-LC1* anti-liver cytosol antibodies type 1

To sum up, pIgG was significantly elevated in children with AIH compared to children with non-AIH LD and HC (Fig. 4a). Of all children with AIH, 72.6% where positive for anti-HIP1R/BSA while 13.4% of non-AIH LD and 6% of HC were positive (*p* < 0.001 vs. AIH, respectively). Children with AISC had anti-HIP1R/BSA levels between AIH and non-AIH LD (Fig. 4a). Polyreactive IgG had comparable sensitivity to ANA and anti-SMA if the cut-off of 1:80 was applied and slightly inferior sensitivity with the cut-off of 1:20 for ANA and anti-SMA while being superior to anti-LKM, anti-LC1 and anti-SLA/LP (Fig. 4b, Suppl. Figure 4). Specificity of pIgG was higher than that of ANA and anti-SMA and lower than that of anti-LKM and anti-SLA/LP, irrespective of the cut-off used for ANA, anti-SMA or anti-LKM. This translated to a significantly higher overall accuracy of pIgG compared to all other autoantibodies (Fig. 4b, Suppl. Figure 3). A post-hoc power of 100% to distinguish AIH (n = 84) from non-AIH LD (n = 119) was calculated based on the overall test fidelity (α-error = 0.05).

## Discussion

As recently published in adults [19], polyreactive IgG from pediatric patients with AIH exhibited increased polyreactivity to multiple protein and non-protein substrates compared to non-AIH LD and HC. Polyreactive IgG had the highest AUC to distinguish AIH from non-AIH LD. As IIF is dependent on the observer, it was done centrally in our center and compared to centrally assessed anti-HIP1R/BSA measurements. With this approach, pIgG was equally sensitive as ANA and anti-SMA while being more specific and having the highest accuracy of all autoantibodies to diagnose or rule-out AIH.

In children, lower titers of autoantibodies are regarded as diagnostic as children are less often positive for any autoantibody in the absence of disease in comparison to adults [13, 14, 27], but nonetheless ANA were more frequently observed in children with Wilson’s Disease and ANA and anti-SMA were frequently present in children with MASLD thereby limiting their specificity if they were used to discriminate AIH from these entities in any unclear hepatopathy [28, 29]. We recently described the quantification of pIgG as a valuable diagnostic tool for AIH in adults with comparable sensitivity to ANA and anti-SMA and higher specificity [19]. Quantification of pIgG provided additional diagnostic value in seronegative AIH and patients with normal IgG in adults. In addition, the ELISA-based assay does not require as much experience and time as the interpretation of IIF does. In the current study, we were able to validate previous findings on adult patients in a large and unique European cohort from well-recognized pediatric centers. Quantification of pIgG provided equal sensitivity to ANA and anti-SMA with the more conservative 1:80 cut-off, slightly lower sensitivity with the 1:20 cut-off but better sensitivity than anti-LKM and anti-SLA/LP. Specificity of pIgG was superior to ANA and anti-SMA but inferior to highly disease specific and rarer anti-LKM and anti-SLA/LP. In children, as recently in adults, we found the highest overall accuracy for the quantification of pIgG and demonstrated the same excellent performance in AIH with normal serum IgG. Unfortunately, given the high sensitivity introduced by the very low cut-offs of 1:20, there was only one child with seronegative AIH. If a more conservative cut-off of 1:80 was used, seven children had seronegative AIH of which five were positive for pIgG. Whilst the small number of children with seronegative AIH precludes any meaningful statistical analysis, the data is in line with the data from adults [19] and is reassuring that there might be additional value for the quantification of pIgG in seronegative AIH, especially as pIgG levels did not differ dependent on the presence or absence of ANA or anti-SMA. Children with AISC had higher levels of pIgG compared to other non-AIH LD subgroups and HC, but clinical presentation, biochemical tests, bile-duct imaging and the current scoring system [6] should sufficiently aid in differentiating these entities. Of note, approximately 20% of children with childhood-onset AIH appear to develop features of biliary disease [30] and the role of pIgG or any other autoantibody to detect autoimmunity that necessitates immunosuppressive treatment in contrast to AISC or PSC may in this case only be answered by serial testing and clinical follow-up in these children. Other groups of interest in daily clinical practice to differentiate from AIH are WD and DILI/herb-induced liver injury. The levels of pIgG appeared reasonably lower in WD, which were included in the hereditary/metabolic subgroup (Suppl. Figure 2b) compared to untreated AIH, however, sample size prevented solid comparison as it did with DILI. Concerning the DILI cases, differentiation from drug-induced autoimmune-like hepatitis that necessitates short-term immunosuppressive treatment is crucial [31]. However, the cases included never needed immunosuppressive treatment until the end of follow-up and spontaneously resolved despite diclofenac, which is associated with drug-induced autoimmune-like hepatitis, being accused as causative drug in one case [31].

Because our study was retrospective, it bears the potential bias of non-standardized sample processing. We performed normalization for storage duration and center background as described [19] to compensate for these factors and allow comparison between children and adults. The heterogeneity of center-dependent test performance of IIF was compensated by centralized performance of IIF. Interestingly, we found pIgG levels to be higher in patients with typical AIH histology in comparison to those with only compatible histology in the training cohort while the analysis in the validation cohort was limited by only three patients having a compatible histology. Whilst this analysis is limited by the retrospective design of the study as well as non-centralized histopathological assessment it warrants further analysis. Overall, the study reflects a real-world setting in which pIgG maintains its superior diagnostic properties.

Unfortunately, a prospectively collected cohort from children using standardized sample processing and longitudinal follow-up, as published for pIgG in adults [19], which would have allowed serial measurements was not available. However, center background and storage duration appeared not to have an effect as high as in adults and normalization even narrowed the gap of AIH and non-AIH LD for other centers (Suppl. Figure 1), thus reducing test performance of pIgG in comparison to non-normalization. Concerning the follow-up, antibody titers have been implicated in disease activity in children and normalization (or very low titers) can precede treatment withdrawal [6, 32]. In adults antibody titers do not define remission due to insufficient data. We demonstrated pIgG to normalize in adults but did not have enough follow-up samples of children to examine it in this study. This further supports study in a subsequent prospective trial with serial sampling which is currently ongoing (NCT05810480).

As expected, we were able to lower the reactivity of pIgG by serial dilution but did not succeed to saturate binding capacities by preincubation with BSA as it is possible for defined autoantigens like LKM [33], supporting the concept of pIgG as a diagnostic test for AIH as described [19]. Differences concerning test interference are not expected in children as compared to adults and are generally hampered by availability of less serum in children.

The concept of pIgG is well in line with current understandings of the pathophysiology of AIH. pIgGs per se arise during class switching from IgM to IgG and during somatic hypermutation in B cells [34, 35]. Antigen-specific helper T cells stimulate B cells not necessarily dependent on the B cell receptor specificity [34]. A recent study found a specific T cell receptor repertoire in AIH patients but no specificity of the B cell receptor repertoire [36]. Hence, hypergammaglobulinemia and polyreactivity of IgG are regarded as an epiphenomenon of an unleashed immune response. A point worthy of note: Even the T cell receptor repertoire is less specific in AIH than in hepatitis C, arguing for a polyreactivity in AIH that is supported by current animal models [36–38].

The identification of immunogenic epitopes targeted by autoantibodies, e.g. CYP2D6_193-212_ facilitated the development of solid-phase immunoassays for autoantibody detection [39]. However, performance of such assays has not been evaluated in large multi-center studies, especially for the diagnosis of AIH in children, given the much rarer occurrence of type 2 AIH as also seen in our study. In general, it is well-known that patients’ IgG can interfere with immunoassays by means of polyreactivity to a variety of protein and non-protein antigens potentially creating false-positive results compromising specificity [40, 41]. Specifically, interference by pIgG is present in diseases with polyclonal hypergammaglobulinemia and severe inflammation, both hallmark features of AIH [27, 40]. In addition, a recent study compared commercially available ELISA for ANA and F-actin against antibody titers derived from IIF [17]. Of note, regarding concordance of IIF and ELISA in this study, up to 80.4% of AIH patients were false-negative in ELISA and up to 60% of AIH patients were false-positive. Concerning the latter, high levels of pIgG appear to be a potential source of interference in ELISA assays measuring IgG, as these react with multiple blocking reagents commonly used[18]. We advocate ruling out polyreactivity prior to the interpretation of positive ELISA test results in untreated AIH patients or to rely on alternative diagnostic methods. Generally, immunoassays are currently regarded as complementary diagnostic tools to provide additional information on findings in IIF [14, 42, 43].

To sum up, pIgG is common in children with untreated AIH and the current study closed the gap of our first description of diagnostic value of pIgG in adults. Demonstrated through the measurement of patients’ IgG reactivity to HIP1R/BSA using an ELISA in a quantified manner, pIgG remains unaffected by the usual autoantibody presence. This may offer supplementary significance for children diagnosed with AIH who have normal IgG levels or are negative for conventional autoantibodies. Considering the superior precision and enhanced specificity of pIgG compared to ANA and anti-SMA, its use may enhance the existing diagnostic arsenal for investigating potential AIH cases. This bolsters diagnosis making, potentially preventing needless treatment or postponement of necessary treatment.

### Supplementary Information

Below is the link to the electronic supplementary material.

Supplementary file1 (PDF 748 KB)

## Data Availability

The data that support the plots within this paper and other findings of this study are available from the corresponding author upon reasonable request.

## Abbreviations

AIH: Autoimmune hepatitis
AISC: Autoimmune sclerosing cholangitis
ALT: Alanine aminotransferase
ANA: Antinuclear antibodies
AST: Aspartate aminotransferase
AU: Arbitrary unit
BR: Biochemical response
BSA: Bovine serum albumin
DILI/HILI: Drug/herb-induced liver injury
HC: Healthy controls
HIP1R: Huntingtin-interacting protein 1-related protein
HSA: Human serum albumin
IgG: Immunoglobulin G
IIF: Indirect immunofluorescence
IR: Incomplete response
ITSN1: Intersectin 1
anti-LC1: Anti-liver cytosol antibodies type 1
anti-LKM: Anti-liver kidney microsomal antibodies
MASLD: Metabolic dysfunction-associated liver disease
MASH: Metabolic dysfunction-associated steatohepatitis
nAU: Normalized arbitrary unit
non-AIH LD: Non-AIH liver diseases
PSC: Primary sclerosing cholangitis
pIgG: Polyreactive immunoglobulin G
anti-SLA/LP: Anti-soluble liver antigen/liver pancreas antibodies
anti-SMA: Anti-smooth muscle antibodies
SR: Spearman rank-correlation coefficient
UBC: Ubiquitin
TBST: TBS with 0.025% Tween Twenty
TF Pierce: ThermoFisher Pierce™ Protein-Free blocking buffer
WD: Wilson’s disease
