# Supplementary material for: Detection of polyreactive immunoglobulin G facilitates diagnosis in children with autoimmune hepatitis
Source: Hepatol Int. 2024 Jul 8;18(4):1214–26. doi: 10.1007/s12072-024-10695-1 (PMC11297808; doi:10.1007/s12072-024-10695-1)
Supplement: Supplementary file 1 — Supplementary file1 (PDF 748 KB) [file 12072_2024_10695_MOESM1_ESM.pdf]

Detection of polyreactive immunoglobulin G facilitates diagnosis in children with autoimmune hepatitis

*Bastian Engel[1, 15], *Jana Diestelhorst[1, 2, 15, &], Katharina Luise Hupa-Breier[1, 15], Theresa Kirchner[1, 15], Nicole Henjes[1, 15], Stephanie Loges[1, 15], Muhammed Yuksel[3, 4, 5], Wojciech Janczyk[6, 15], Claudine Lalanne[7, 15], Kalliopi Zachou[8, 9, 15], Ye H. Oo[10, 15], Jérôme Gournay[11], Simon Pape[12, 15], Joost PH Drenth[12, 15], Amédée Renand[13], George N. Dalekos[8, 9, 15], Luigi Muratori[7, 15], Piotr Socha[6, 15], Yun Ma[3], Cigdem Arikan[4, 14], Ulrich Baumann[2, 15], Michael P. Manns[1, 15], Heiner Wedemeyer[1, 15], #Norman Junge[2, 15], #Elmar Jaeckel[1, 15, +], #Richard Taubert[1, 15]

* authors share first authorship

# authors share last authorship

Table of contents

METHODS.. 2

FIGURES.. 7

TABLES 10

REFERENCES 19

Methods

Quantification of polyreactive immunoglobulin G

Patients’ serum samples from Hannover Medical School were cryo-conserved at below -20°C. Serum samples from external centers were cryo-conserved according to local protocols and sent frozen to Hannover Medical School for centralized quantification of pIgG. Samples were pseudonymized for autoantibody-testing and observers were blinded to any clinical information.

Quantification of pIgG using an ELISA to quantify reactivity to a peptide and BSA as blocking agent was performed as published[1]. In short, 0.01 µg of HIP1R fragment, 0.1µg of intersectin 1 (ITSN1) fragment or 0.01 µg of ubiquitin (UBC) per well was bound to 96 well ELISA plates over night at 4 °C. Plates were blocked with TBS and 5 % BSA for 30 minutes. Plates were washed with TBS with Tween20® 0.05 % (TBST) once. Serum samples were diluted 1:101 (v/v) in TBS and 5 % BSA and 100 µl per well were added to the ELISA plate and incubated for two hours. Plates were washed three times with TBST and incubated with a secondary rabbit anti-human anti-IgG antibody labeled with horseradish peroxidase for 30 minutes. Three washing steps with TBST were performed and 3, 3', 5, 5' tetramethyl benzidine (BioLegend, San Diego California) was added for 30 minutes for color reaction. Reaction was stopped with sulfuric acid. Optical density was read at 450 nm using an ELISA reader (Tecan Sunrise-Basic, Grödig, Austria). Sera of five patients with

gradual increase in pIgG reactivity were measured in every experiment and used to compute a standard curve. Arbitrary units (AU) were calculated from the equation of the standard curve. Measurements were performed in Hannover, Germany.

As different AU dependent on center and storage duration were demonstrated, a normalization for these factors (referred to as normalized AU (nAU)) was performed as published[1].

Testing of different blocking reagents

96-well plates (Maxisorp, Nunc, Denmark) were blocked with 300 µl per well of 5 % BSA (Sigma-Aldrich, Germany) in TBS, 20 g/l HSA (Shire, Germany) diluted to a final concentration of 5 % in TBS, 0.2 % Casein (Sigma-Aldrich, Germany) in TBS, 1 % nonfat dried milk powder (AppliChem GmbH, Germany) in TBS, 2 % Tween® 20 (Sigma-Aldrich, Germany) in TBS, SmartBlock™ (Candor, Germany) or Pierce™ Protein-Free Blocking Buffer (ThermoFisher Scientific Inc, USA) respectively. The ELISA was continued as described in the Methods section.

Liquid-Liquid Preincubation

Patient sera were diluted 1:250 (v/v) in 20 % BSA in TBS. Diluted samples were stored for preincubation at 4 °C over night. BSA preincubated samples were diluted 1:4 (v/v) due to prior dilution in TBS. These samples were added to the ELISA after blocking of the 96-well plates (100 µl/well) as described in the Methods section.

Immunofluorescence testing

IFT was performed by experienced technicians using the recommended methodology of the guidelines issued in 2004 by the Committee for Autoimmune Serology of the International Autoimmune Hepatitis Group[2]. Samples were pseudonymized for autoantibody-testing and observers were blinded to any clinical information. ANA, anti-SMA, anti-LKM and anti-LC1 were detected by IIF on sections of frozen rodent liver, stomach and kidney sections. Briefly, a commercial rodent multi-organ substrate panel (kidney, liver and stomach) was used (LKS Rat wrapped Standard Kit, Aesku.Diagnostics GmbG & Co. Wendelsheim, Germany). The sera were diluted, starting with a dilution of 1:20 up to 1:160, and applied to the slide to cover the entire tissue section and allow binding of the autoantibodies to the substrates. After washing, the sample was exposed to a second fluorochrome-labeled antibody. Finally, once washed again, the slides were examined under fluorescence microscope (Olympus BX60 Microscope, Evident Europe GmbH, Germany), and the antibody staining pattern was evaluated and interpreted accordingly to the guidelines[2].

ELISA testing

An in-house ELISA was performed as published[3,4]. Samples were pseudonymized for autoantibody-testing and observers were blinded to any clinical information. Briefly, antibodies from defined anti-SLA or anti-LKM1 indicator sera were coated overnight in a volume per well of 50µl at room temperature in microtiter plates (Dynatech, el Paso, Texas for anti-LKM1, and Maxisorp, Nunc,

Denmark for SLA). The supernatants were removed and after a washing step respective antigens were added. For the generation of the antigens rat liver was homogenized and centrifuged for 15 minutes at 3000 rpm. The pellet was discarded and the supernatant was centrifuged at 8500 rpm for 15 minutes. The pellet was discarded. The supernatant was further centrifuged for 60 min at 50000 rpm, antigens for LKM were collected from the pellet and antigens for SLA were collected from the supernatant and were added to the respective ELISA. All antigens were added at a concentration of 100 µg/ml and incubated for one hour at room temperature. Patient samples were diluted 1:10 in PBS + 10 mM EDTA and added to the microtiter plates following two washing steps. Incubation was done for one hour at room temperature. Following three additional washing steps, avidin-peroxidase and sodium perborate dissolved in citrate buffer were added to microtiter plates. The photometric reaction was stopped after five minutes, and the absorbance was measured. The percentage of inhibition of the indicator serum to its respective autoantigen was used as a surrogate for the antibody titer.

Statistical analysis

Statistical analysis was performed using SPSS (version 27, SPSS, Inc., Chicago, IL), GraphPad Prism (version 10.0.2 (232), GraphPad Software Inc., La Jolla, CA), MedCalc software (version 19.4.1, MedCalc Software Ltd, Ostend, Belgium) and easyROC package (version 1.3.1)[26] in RStudio (2021.09.0+351 "Ghost Orchid" Release, RStudio, PBC, Boston, MA). The Mann-Whitney U test was used to compare quantitative data between two groups and the Kruskal-Wallis test was used for more than two groups with

Bonferroni post-hoc test. The Fisher's exact test was used to compare categorical variables. Correlation analyses were calculated with Spearman's rank correlation. The AUC and the Youden's Index were used to guide identification of respective cut-off values. AUCs were compared using DeLong's test[27].

Accuracy of diagnostic test was calculated as (true positive+true negative)/total number. Sensitivities and specificities were compared with the McNemar test. Overall accuracies were compared by the comparison of the 95 % CI. P-values below 0.05 (two-tailed) were considered significant in all analyses.

Figures

Supplemental Figure 1

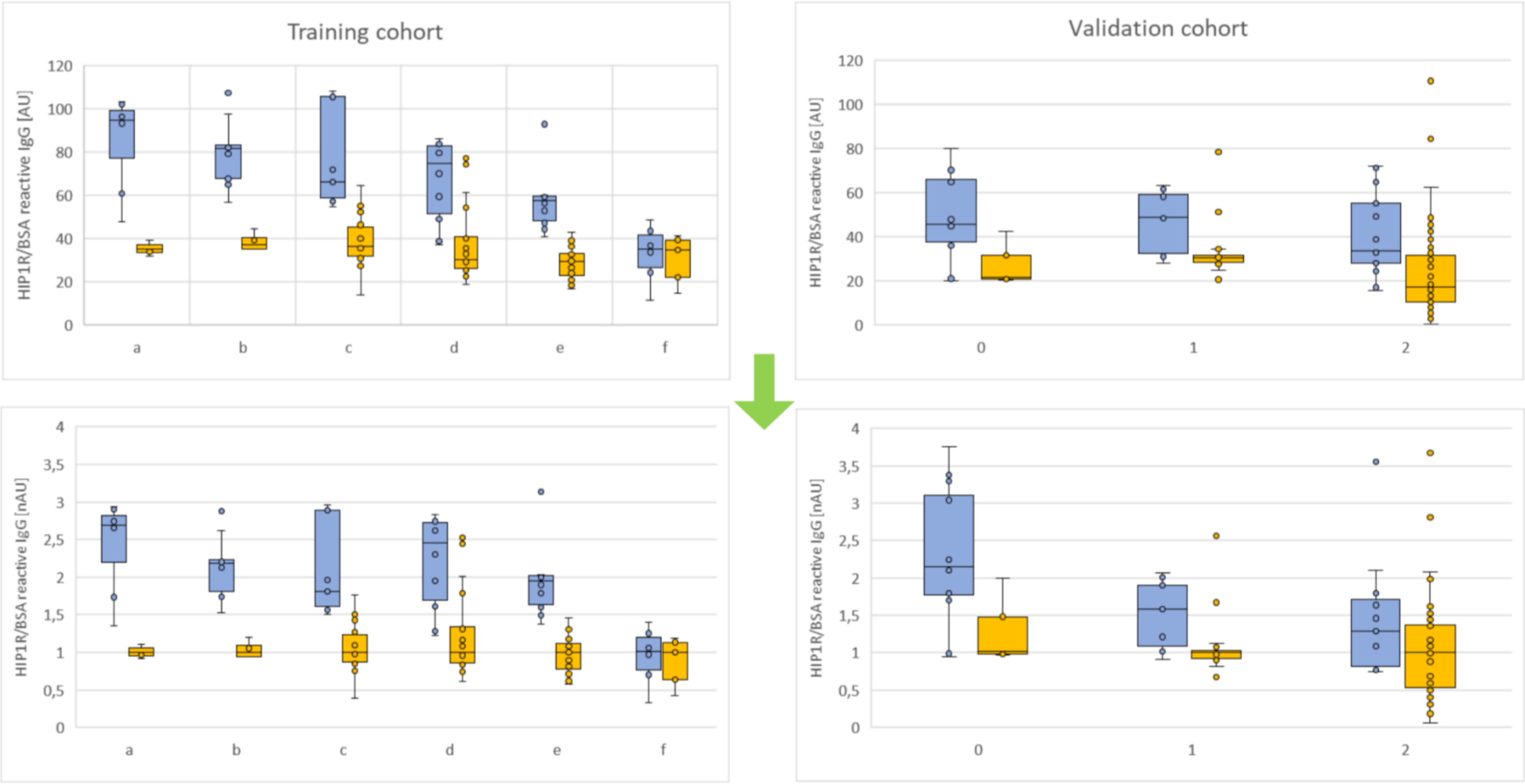

0: 1997 - 2009; 1: 2010 - 2016; 2: 2017 - 2022

a: 1994 - 2002; b: 2003 - 2005; c: 2007 - 2009;
d: 2010 - 2012; e: 2013 - 2015; f: 2016 - 2018

AIH   non-AIH LD + HC

Supplemental figure 1: Normalization to center background and storage duration

Boxplots of anti-HIP1R/BSA reactive IgG, quantified using reference samples to calculate a standard curve. Depicted in arbitrary units (AU) (median, interquartile range) (upper panels). Lower panel shows normalized arbitrary units (nAU) after normalization to center-background of non-AIH liver disease (non-AIH LD) and healthy controls (HC) (median, interquartile range). HIP1R: huntingtin-interacting protein 1-related protein; BSA: bovine serum albumin.

Supplemental Figure 2

(A)

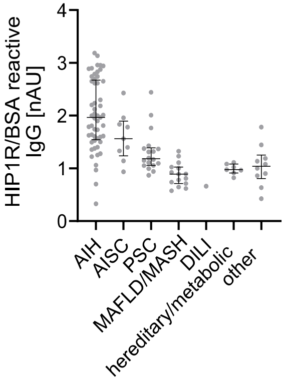

Training	
Comparison	p-value
AIH –AISC	1.000
AIH –PSC	0.009
AIH –MAFLD/MASH	<0.001
AIH –hereditary/metabolic	0.001
AIH –other	<0.001
AISC –PSC	1.000
AISC –MAFLD/MASH	0.035
AISC –hereditary/metabolic	0.497
AISC –other	0.777

(B)

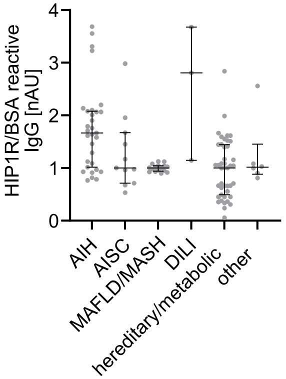

Validation	
Comparison	p-value
AIH –AISC	0.971
AIH –MAFLD/MASH	0.027
AIH –hereditary/metabolic	<0.001
AIH –other	1.000
AISC –MAFLD/MASH	1.000
AISC –hereditary/metabolic	1.000
AISC –other	1.000

Supplemental figure 2: Anti-HIP1R/BSA reactive IgG in children with autoimmune hepatitis (AIH) compared to subgroups of non-AIH liver disease

Anti-HIP1R/BSA reactive IgG in children with AIH, AISC and non-AIH liver diseases (non-AIH LD) in the training cohort (a) and validation cohort (b). Data are shown as median and interquartile range. The tables provide respective p-values for post-hoc two-group comparisons (Kruskal Wallis test with Bonferroni's post-hoc test). Comparisons with DILI were not calculated because of the small group size. All other comparisons were not significant in post-hoc testing. AISC: autoimmune sclerosing cholangitis; NAFLD: non-alcoholic fatty liver disease; NASH: non-alcoholic steatohepatitis; PSC: primary sclerosing cholangitis.

Supplemental Figure 3

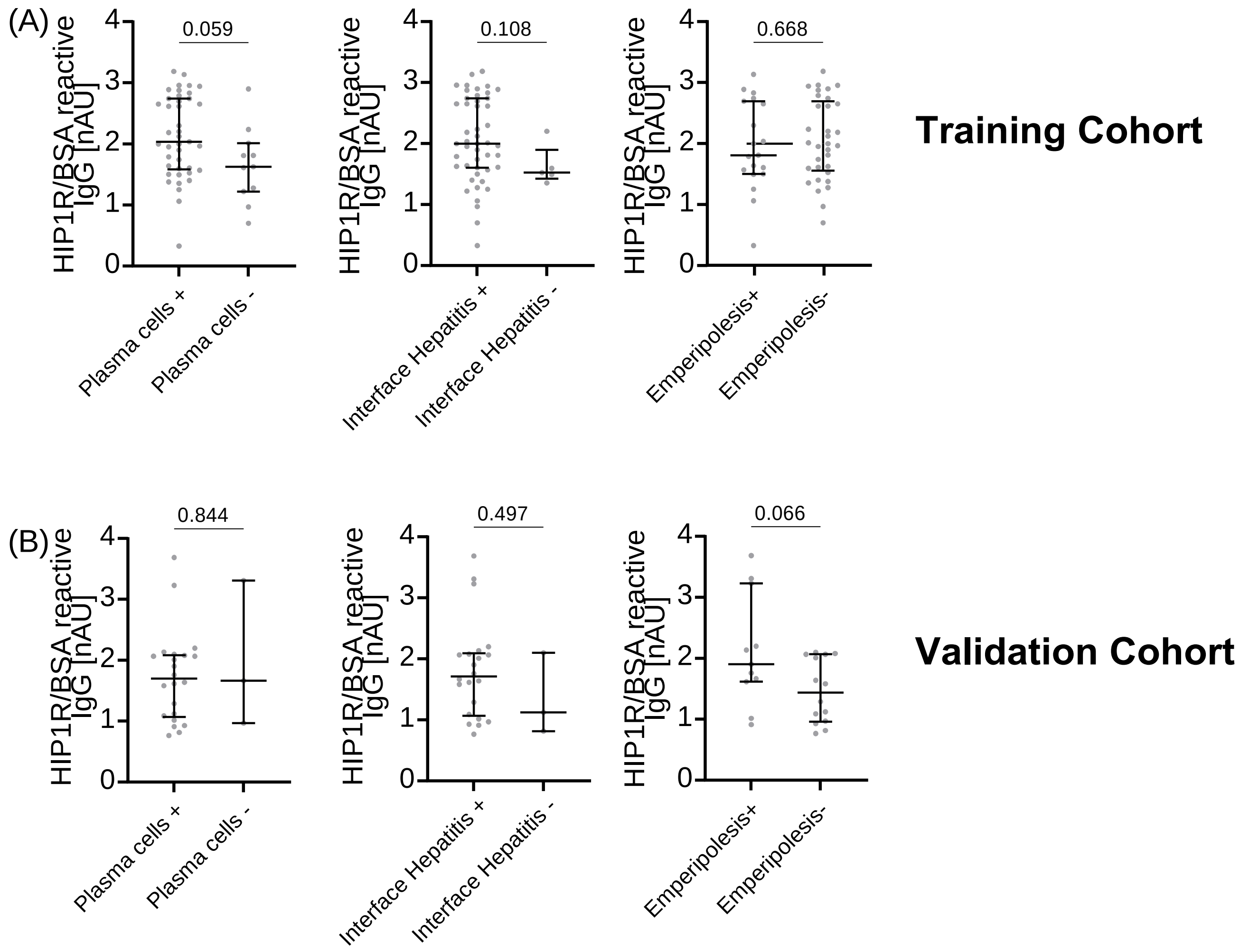

Supplemental figure 3: Levels of pIgG in children with or without specific histological features

Comparison of pIgG levels between children with (Plasma cells +) and without plasma cells (Plasma cells -), interface hepatitis (Interface Hepatitis + or – respectively) or Emperipolesis (Emperipolesis + or – respectively) in the training (A) and validation cohort (B). (Mann-Whitney U test. HIP1R: huntingtin-interacting protein 1-related protein; BSA: bovine serum albumin; ANA: antinuclear antibodies; anti-SMA: anti-smooth muscle antibodies; nAU: normalized arbitrary units.

Supplemental Figure 4

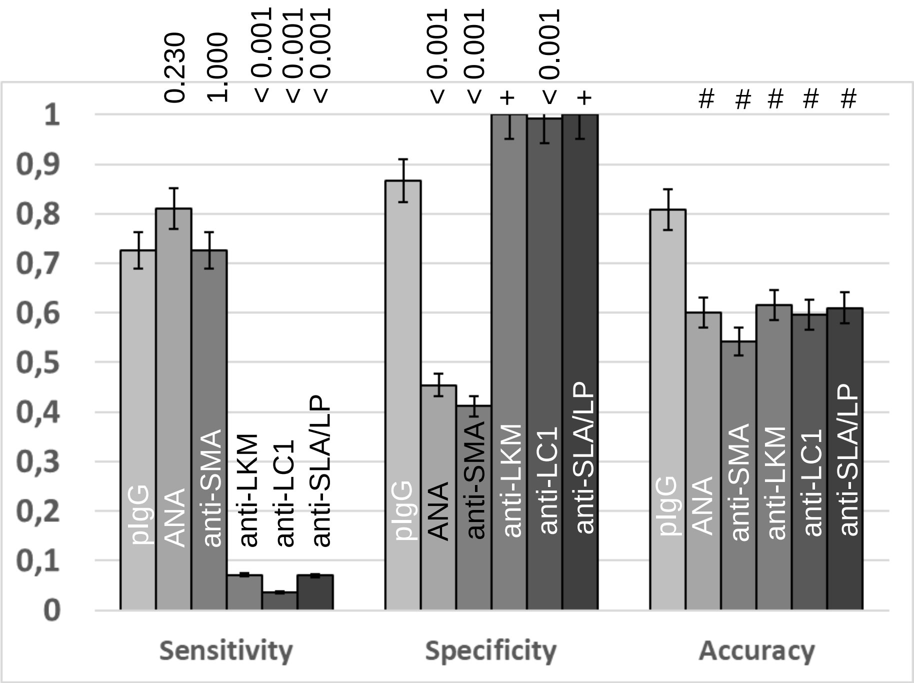

Supplemental figure 4: Diagnostic properties of pIgG compared to conventional autoantibodies

Diagnostic properties of polyreactive IgG (pIgG) in comparison to conventional autoantibodies (cut-off 1:80 for ANA, anti-SMA and anti-LKM) of AIH (McNemar test in case p-value is shown; + McNemar test not applicable, superiority to pIgG by comparison of 95 % CI; # inferiority to pIgG by comparison of 95 % CI) HIP1R: huntingtin-interacting protein 1-related protein; BSA: bovine serum albumin; ANA: antinuclear antibodies; anti-SMA:

anti-smooth muscle antibodies; anti-SLA/LP: anti-soluble liver antigen/liver-pancreas antibodies; anti-LKM: anti-liver kidney microsomal antibodies, anti-LC1: anti-liver cytosol antibodies type 1.

Tables

Supplemental table 1: Clinical data of healthy control patients

	**Training Cohort**	**Validation cohort**	
	**HC**	**AIH on treatment**	**HC**
**Number**	34	13	16
**Age (median (range))**	11 (3 - 16)	14 (1 - 18)	12 (4 - 17) (n=15)
**Female sex n (%)**	23 (67.6)	8 (61.5)	6 (37.5)
**AST, median [times upper limit of normal] (range)**	0.9 (0.4 - 1.7) (n=31)	0.6 (0.3 - 1.3) (n=12)	0.5 (0.3 - 3.2) (n=5)
**ALT, median [times upper limit of normal] (range)**	0.4 (0.2 - 1.6) (n=30)	0.6 (0.3 - 2.1) (n=12)	0.7 (0.3 - 4.7) (n=5)
**IgG, median [times upper limit of normal] (range)**	0.8 (0.5 - 1.0) (n=16)	0.7 (0.5 - 0.8) (n=6)	na

Supplemental table 2: Clinical data of the test cohort with available data on HIP1R/BSA, ITSN1/BSA and UBC/BSA reactive IgG

	untreated AIH	non-AIH liver disease
Number	47	46
Age (median (range))	12 (3 - 17)	12 (0 - 17)
Female sex n (%)	35 (74.5)	14 (30.4)
AST, median [times upper limit of normal] (range)	10.6 (1.1 - 114.1)	1.8 (0.7 - 50.9) (n=45)
ALT, median [times upper limit of normal] (range)	11.5 (1.0 - 84.9)	2.2 (0.3 - 40.2) (n=45)
IgG, median [times upper limit of normal] (range)	1.7 (0.5 - 6.1)	0.8 (0.5 - 1.7) (n=40)
seronegative AIH, n (%)	0 (0)	na

AIH-1, n (%)	44 (93.6)	na
AIH-2, n (%)	3 (6.4)	na

Supplemental table 3: AUCs and respective cut-offs for anti-HIP1R/BSA reactive IgG in the training cohort with and without inclusion of AISC

**Without AISC**					
AUC	*p*	*95 % CI*	*Cut-off*	*number AIH cases*	*number non-AIH-LD cases*
0.900	< 0.001	0.835 - 0.965	1.5	53	51
**With AISC**					
AUC	*p*	*95 % CI*	*Cut-off*	*number AIH&AISC cases*	*number non-AIH-LD cases*
0.890	< 0.001	0.826 - 0.954	1.4	61	51

Supplemental table 4: Diagnostic test performance of different antibodies to distinguish AIH from non-AIH liver disease with higher cut-off for ANA, anti-SMA and anti-LKM1

	Sample number	cut off	Sensitivity	95 % CI	p vs anti-HIP1R/BSA (McNemar)	Specificity	95 % CI	p vs anti-HIP1R/BSA (McNemar)	Accuracy	95 % CI
anti-HIP1R/BSA	104	≥ 1.5 nAU	0.793	0.659 - 0.892		0.922	0.811 - 0.978		0.856	0.773 - 0.917
ANA	104	≥ 1:80	0.793	0.659 - 0.892	1.000	0.608	0.461 - 0.742	< 0.001	0.702	0.604 - 0.788
anti-SMA	104	≥ 1:80	0.698	0.557 - 0.817	0.302	0.529	0.385 - 0.671	< 0.001	0.615	0.515 - 0.709
anti-LKM	104	≥ 1:80	0.057	0.012 - 0.157	< 0.001	1.000	0.930 - 1.000	na	0.519	0.419 - 0.618
anti-LC1	104	pos.	0.038	0.005 - 0.130	< 0.001	0.980	0.896 - 1.000	0.375	0.500	0.400 - 0.600
anti-SLA	88	pos.	0.065	0.014 - 0.179	< 0.001	1.000	0.916 - 1.000	na	0.511	0.403 - 0.620
anti-HIP1R/BSA	99	≥ 1.5 nAU	0.613	0.422 - 0.782		0.824	0.712 - 0.905		0.758	0.661 - 0.838
ANA	99	≥ 1:80	0.839	0.663 - 0.946	0.092	0.338	0.228 - 0.463	< 0.001	0.495	0.393 - 0.597
anti-SMA	99	≥ 1:80	0.774	0.589 - 0.904	0.180	0.324	0.215 - 0.448	< 0.001	0.465	0.364 - 0.568
anti-LKM	99	≥ 1:80	0.097	0.020 - 0.258	< 0.001	1.000	0.947 - 1.000	na	0.717	0.618 - 0.803
anti-LC1	99	pos.	0.032	0.001 - 0.167	< 0.001	1.000	0.947 - 1.000	na	0.697	0.597 - 0.785
anti-SLA	48	pos.	0.091	0.002 - 0.413	0.375	1.000	0.905 - 1.000	na	0.792	0.650 - 0.895

Supplemental table 5: Characterization of DILI cases in the validation cohort

causative Agent	Age	sex	ALT [xULN]	AST [xULN]	AP [xULN]	gGT [xULN]	Bilirubin [xULN]	IgG [xULN]	INR	hepatic encephalopathy	Autoantibodies positive	pIgG positive	histology	Immunosuppressive Treatment @ 6 months	immunosuppressive treatment at end of follow up	duration of follow up [years]
diclofenac	17	f	216,6060606	152,4375	0,632478632	1,424242424	3,222222222	na	2,19	no	yes (ANA 1:80, anti-SMA 1:40)	no	Florid perivenular necrosis with neutrophilic and lymphocytic inflammation. Moderate portal mixed inflammation. Preserved (normal) biliary structure	no	no	3,5
amoxiklav	4	m	37,35897436	1,865384615	0,755223881	22,94444444	1,955555556	na	1,19	no	yes (ANA 1:80, anti-SMA 1:80)	yes	portal inflammation with lymphocytes, neutrophils and eosinophils. Focal lymphocytic cholangitis. Bridging perivenular necrosis with dens lymphocytic inflammation	no	no	3
flagyl-amoxclav-paracetamol	1	m	29,9	36,46067416	0,490405117	20,58823529	3,955555556	0,549375	1,07	no	yes (ANA 1:160, anti-SMA 1:160)	yes	severe portal inflammation with eosinophils, some neutrophils and lymphocytes. Portal bridging necrosis, multiple fokal necrosis. Ductular proliferation.	no	no	3

References

1. Taubert R, Engel B, Diestelhorst J, et al. Quantification of polyreactive immunoglobulin G facilitates the diagnosis of autoimmune hepatitis. *Hepatology*. 2022;75(1):13-27. doi:10.1002/hep.32134

2. Vergani D, Alvarez F, Bianchi FB, et al. Liver autoimmune serology: a consensus statement from the committee for autoimmune serology of the International Autoimmune Hepatitis Group. *J Hepatol*. 2004;41(4):677-683. doi:10.1016/j.jhep.2004.08.002

3. Manns M, Kyriatsoulis A, Gerken G, Staritz M, Meyer KH, Büschenfelde Z. CHARACTERISATION OF A NEW SUBGROUP OF AUTOIMMUNE CHRONIC ACTIVE HEPATITIS BY AUTOANTIBODIES AGAINST A SOLUBLE LIVER ANTIGEN. *Lancet*. 1987;329(8528):292-294. doi:10.1016/S0140-6736(87)92024-1

4. Kanzler S, Weidemann C, Gerken G, et al. Clinical significance of autoantibodies to soluble liver antigen in autoimmune hepatitis. *J Hepatol*. 1999;31(4):635-640. doi:10.1016/S0168-8278(99)80342-0
